# The Subjective Experience of Using Medications: What We Know and the Paths Forward

**DOI:** 10.3390/pharmacy9010050

**Published:** 2021-03-02

**Authors:** Yone de Almeida Nascimento, Djenane Ramalho-de-Oliveira

**Affiliations:** 1 Center for Pharmaceutical Care Studies [Centro de Estudos em Atenção Farmacêutica–CEAF-UFMG], College of Pharmacy, Universidade Federal de Minas Gerais, Belo Horizonte CEP 31270-901, Minas Gerais, Brazil; droliveira@ufmg.br; 2 Centro Universitário Newton Paiva, Belo Horizonte 30431-189, Minas Gerais, Brazil

**Keywords:** medication experience, medication use, phenomenology, Merleau-Ponty

## Abstract

Medications can cause bodily changes, where the associated benefits and risks are carefully assessed based on the changes experienced in the phenomenal body. For this reason, the phenomenology of Merleau-Ponty is an important theoretical framework for the study of experience related to the daily use of medications. The aim of this study was to discuss the contribution of a recently developed framework of the general ways people can experience the daily use of medications—*resolution, adversity, ambiguity, and irrelevance*—and present reflections about the little-understood aspects of this experience. However, some issues raised throughout this article remain open and invite us to further exploration, such as (1) the coexistence of multiple ways of experiencing the use of medications, by the same individual, in a given historical time; (2) the cyclical structure of this experience; (3) the impact of habit and routine on the ways of experiencing the daily use of medications; and (4) the contribution of the concept of existential feelings to this experience and its impact on patients’ decision-making. Therefore, the experience with the daily use of medications is a complex and multifaceted phenomenon that directs the decision-making process of patients, impacting health outcomes.

## 1. Introduction

Merleau-Ponty innovated when he placed perception and the body as central points of his philosophical proposal [1,2]. Embodiment is a different way of thinking about and knowing human beings, one that is in contrast to the usual Western thinking of mind and body as separate (dualism) [3]. The distinction made by Merleau-Ponty between the *body object*, the physical body of objective science, and the *phenomenal or lived body*, responsible for experience, is essential in his work. As *beings-in-the-world*, we apprehend, interact with, and understand the world through a flux of experiences that are anchored in the *phenomenal body* [4,5]. This is embodied consciousness, which simultaneously engages and is engaged in the surrounding world [6]. In a nutshell, *phenomenal body* refers to how we place ourselves in the world through our bodies, where “the body is the vehicle of being in the world, and having a body is, for a living creature, to assemble with a definite environment, to identify oneself with certain projects and be continually committed to them” [4] (p. 122). Being an embodied subject also means being situated in the world, i.e., being affected by social, cultural, political, and historical forces [2,3,4].

In our daily lives, we do not stop to consider the processes underway in our bodies during existence and we do not notice the multiplicity of actions and the necessary expertise needed to execute mundane activities [7,8]. However, the ease with which we carry out these activities disappears in illness because the ability to carry them out is lost and our attention is drawn to the part of us that is not functioning adequately. In this stage, there is a break in the harmony between the *object body* and the *phenomenal body*, whose meaning goes beyond mere mechanical dysfunction of a bodily subsystem, affecting our being-in-the-world [6]. It is a presence that modifies life, transforming how we experience our body, react, and carry out tasks. This disturbance in the relationship between the body and the world can deeply disturb one’s existence [1,2]. The thoughts of Merleau-Ponty allow us to access knowledge that arises from the corporeality of individuals with chronic diseases [1,2].

Medications can cause bodily changes, both positive and negative, and the associated benefits and risks are carefully assessed based on the changes experienced in the body [9]. Nascimento et al. [10] believed that by associating themselves with physical symptoms or symbolizing the disease, medications are capable of changing the phenomenal body. Therefore, analysis based on Merleau-Ponty’s phenomenology is an important theoretical framework for the study of experiences related to the daily use of medications [11,12]. 

It is worth emphasizing that the subjective experience of patients using medications has the potential to influence their decisions about treatment and, consequently, health outcomes [8]. Nascimento et al. [10] highlighted the need to *operationalize* the concept of the experience of using medications, with the goal of contributing to teaching and clinical practice, given that understanding this experience can help professionals identify and solve drug-related problems (DRP) [13]. One of the practical justifications for trying to understand the meaning of medications for patients is that this knowledge contributes to ensuring the greater effectiveness of interventions and improved health outcomes [14].

Currently, it is increasingly difficult for patients with chronic diseases to manage drug treatment, which has become more and more complex [15]. Several studies with different theoretical frameworks have tried to understand the experience of daily medication use, always focusing on positive or negative experiences [9,15,16,17,18,19,20,21,22,23,24,25,26,27,28,29,30,31,32,33,34,35]

To this discussion, Nascimento et al. [10] added a theoretical construction of the general ways people can experience the daily use of medications, which these authors called *resolution*, *adversity*, *ambiguity*, *and irrelevance* (Table 1). Gutheil [36] stated that we no longer have the comfortable illusion that prescribing medications and talking about them are uncomplicated processes. 

For this reason, the aim of the present manuscript was to discuss the contribution of Merleau-Ponty’s theoretical framework, along with the work developed by Nascimento et al. [10,11], in order to incite reflections about the little-understood aspects of the patient experience when using medications in daily life.

## 2. Development

**Figure 1:**
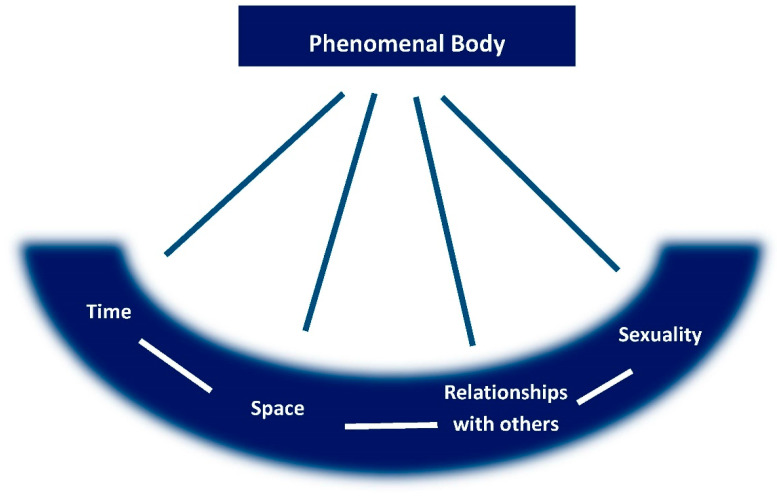
Essential structures of experience.

The effect caused by medications on the phenomenal body modifies the experience of the essential structures of existence (Figure 1), such as social (or other) relationships, insertion in a given context (space), the possibility of living in the present in a fluid way and having hope for the future (time), being shaped by choices (sexuality), and ways to experience the use of medications, creating unique experiences [11]. In a phenomenological philosophy, the fundamental existential categories are implied in the constitution of human experience. According to Merleau-Ponty, we live as embodied subjects who experience the world and its existential categories (time, space, relationship with others, and sexuality) via our lived or phenomenal body [3,4]. Time cannot be understood outside the human being; all of human experience is grounded in time and there can only be present, past, and future time because there are subjects capable of experiencing them in this way [4]. Being-in-the-world also refers to a spatial existence and the phenomenal body is the spatial and temporal center around which the world is organized, the medium through which we exist [4]. In the social world, our relationship with others is a permanent field or dimension of existence and we cannot exist in everyday life without continually interacting and communicating with others [4]. Finally, sexuality can be understood as a certain type of energy that pulsates and leads us to launch ourselves into life situations and must not be confused with an exclusively genital factor. It branches out through all personal existence and can be conceived of as an existential necessity; it is part of the general existence of humans and is inseparable from it [4].

### 2.1. Changes in the Physical and Phenomenal Body 

In illness, the presence of physical symptoms generates the experience of loss of control; familiar body sensations are replaced by other strange and negative ones [6,8]. However, there is a creative reaction to loss, which is the process of adaptability, when people discover new ways to carry out tasks or when they experience well-being in the context of illness, cope with loss, fight for normalcy, and avoid situations that can aggravate manifestations of the disease [30], i.e., “sick, but well” [7]. In this regard, medications can be considered a powerful tool to help control disease [31,37,38]. By re-establishing the unit of the phenomenal body changed by the disease, they contribute to the experience of *resolution* [10]. As a result of the desire to control their illness and have a better life, some patients accept medications unconditionally [33]. 

Nascimento et al. [10] suggested that the role of medications goes beyond controlling physical symptoms; these compounds allow individuals to carry out their daily tasks and move on [30]. This is why, despite difficulties with the medications, patients with schizophrenia use neuroleptics in the hope of recovery and to avoid symptoms of the disease and normalize life, to *go about things as usual* [17]. For patients with diabetes, the use of medications guarantees the continuity of life and silencing the disease, halting manifestations of its acute or chronic complications, and contributing to the experience of *resolution* [10,22]. 

However, for an experience with medication use to be a *resolution,* in addition to solving the problem for which it was indicated, it cannot cause new problems. Moreover, it cannot generate exaggerated concern about the risk inherent to medications [10,16]. 

The pharmacological actions of medications, by causing actual adverse reactions or due to the fear of intolerable treatments, can contribute to the embodied experience being perceived as negative by some individuals [30,31], which was called *adversity* by Nascimento et al. [10]. Adverse reactions are unpleasant, frequent, severe, and unpredictable [19], transforming patients’ bodies into a potential source of awkwardness [29], causing fear, mistrust, and insecurity [19,29,30,32]. They are capable of breaking the tacit certainty that we have in our bodily capacities, which allow us to do what is necessary without second thoughts [7]. For example, patients undergoing treatment for schizophrenia report that the presence of adverse motor reactions caused by neuroleptics turns the body into a source of embarrassment, or in the best-case scenario, an *unpredictable ally*, generating a sense of insecurity. The body loses its transparency, affecting these patients’ *being-in-the-world*. Furthermore, patients feel different from other individuals, even when the disease is under control, eliciting the experience of stigma [17]. 

The sensation of control and predictability are positive aspects that are related to the experience of using medications to control diabetes [31], and for patients who use the anticoagulant warfarin, this is expressed by the constant monitoring of the international normalized ratio (INR) [24], contributing to the experience of *resolution*. 

In contrast, the requirement of constant monitoring to avoid potentially strong reactions is a source of stress for some patients [17]. They report confusion about signs of the disease vs. adverse reactions [17,19], or even signs of aging, and this increases feelings of aversion, dependence, and fear toward medication therapy [15]. Moreover, the instability of glucose levels and the difficulty in achieving ideal control in patients with type 2 diabetes *mellitus* who use insulin is experienced negatively. Many individuals report feeling embarrassed because they are not able to keep their glucose levels under control [21]. 

In *ambiguity*, the elements responsible for the experiences of *resolution* and *adversity* appear once again but are delicately anchored in the cost–benefit relationships that are intrinsic to the use of these products. If the bodily effects are seen as acceptable and the benefits of the medications are perceived by patients as important, they can accept the adverse reactions as compensation [9]. Those who use antidepressants express the wish that one day they will no longer need to resort to them, but they also fear the consequences of discontinuing their use, experiencing conflict between the fear of relapse and the fear of dependence [10,19,20,39]. *Ambiguity* seems to be the essence of the experience of medication use for some patients [10,23,31,32].

Finally, there is a difference between the illness experience and organic disease, which is based on the difference between the body as a subject and the body as an object, respectively [7]. The construction of an identity that is consistent with the experience of the disease, which is necessary for the changes required within the scope of health care, does not arise from the diagnosis of chronic disease, but from the language expressed in the bodies [40]. When the absence of symptoms associated with the disease does not lead to the incorporation of the diagnosis as illness, medications are then directed only to the treatment of organic disease, providing the experience of *irrelevance*, as verified in asymptomatic patients using antihypertensive drugs or hormone replacement therapy [10]. 

One of the most relevant contributions of the work developed by Nascimento et al. [10] was the understanding that the same individual can experience the daily use of medications in different ways at the same time in life, depending on the disease and the drug in question. However, is this a one-off finding, or are the multiple ways of experiencing the use of medications part of the essence of this experience? We believe that the answer to these questions has profound implications for clinical practice. 

#### 2.1.1. Medications and Life: Intrusion or Normalization?

Several studies have demonstrated the constant effort that patients make to minimize the intrusion of disease into their lives and to try to be normal within the context of the abnormality caused by the disease [20,24].

For patients using drugs to control HIV/AIDS, the use of medications has been reported as a slow and painful process, not only because of the number of pills but also because of the complexity of the information given to them by professionals to ensure the use of the prescribed therapeutic schemes [25]. Mohammed et al. [33] called this impact the *burden of medication use*, contributing to the experience of *adversity.*

Sav et al. [29] argued that health professionals have little appreciation of the work associated with the management of chronic diseases. They also claimed that there is a lack of tools to identify individuals who are overwhelmed by the burden of adhering to complicated treatment routines. Finally, they affirmed that although self-care is acclaimed as a solution for the prolonged management of chronic conditions by health professionals and providers, this perspective is not shared by all patients and their caregivers. For these individuals, self-care can become a significant burden [29].

Ellis and Welch [41] affirmed that drug management is a complex process in which patients adopt a set of behaviors in three interrelated contexts: medical appointments, pharmacy visits, and day-to-day management. The authors identified twenty behaviors that are required for the daily use of medications, which include communicating with health professionals, seeking information, creating a list of medications in use, keeping prescriptions within reach, seeking out prescribed medications, creating and establishing routines, preparing and administering doses correctly, performing self-monitoring, adopting strategies to avoid forgetting doses, performing risk assessments based on personal experiences, making decisions about the use of medications, and managing adverse reactions. The numerous activities required to use medications [17,31,41] require the acquisition of new routines, habits, and skills. Routines begin to revolve around the preparation and intake of these drugs, generating a significant impact on how these individuals organize their daily lives [31]. Routines can be understood as strategic behaviors that can be conscious or unconscious [42]. They are also pre-reflective and products of habit [4].

Haslbeck and Schaeffer [15] indicated that health professionals provide information about the importance and the correct way of using drugs but fail to help patients integrate them into their daily lives, which are anchored in existing routines, such as meals. However, there are some negative aspects related to the development of routines. Although necessary, they are easily disturbed and can generate the risk of noncompliance. Moreover, automatism may cause patients to forget whether they took a drug, increasing the risk of drug toxicity [15]. Furthermore, changes in the time of sleep or meals are perceived by patients as an interruption in the natural flow of their lives and like they are losing control [19,33]. Medications interrupt the silent experience of life [31] and break the integrity of the phenomenal body because they require the acquisition of new habits, which contributes to the experience of *ambiguity* in relation to the daily use of medicines [10].

However, routines are also considered essential because they allow for the management of multiple and competing demands of life, enabling the coordination of activities over time, without forgetting important aspects of care [14,24,41], which contribute to the experience of *resolution* [9]. In this regard, routines can be positive when they represent synchrony between individuals and their treatment because, in the words of Merleau-Ponty, “My body is there where it has something to do” [4] (p. 336). According to this philosopher, we only pay attention to what concerns us at a certain moment, requiring attention and effort.

Therefore, from this perspective, it is necessary to have a more thorough understanding of the impact of habit and routines on how patients experience the daily use of medications, whether through *resolution*, *adversity*, or *ambiguity*.

#### 2.1.2. What Is a Medication? The Various Symbols That Permeate a Scientific and Technological Product

Pellegrino [43] stated that humans are capable of creating symbols from everyday objects and experiences, and that something is symbolic when it implies more than its immediate and obvious meaning. He affirmed that few human experiences are as universal and have such symbolic connotations as the act of prescribing and ingesting medication.

Technologies in the medical field, such as catheters for the application of medications [2], as well as diseases [2,40,44], are able to change the phenomenal body, even in the absence of changes in the physical body. The problem does not relate to technology itself, but to objectification [44], i.e., individuals perceive their bodies as “broken machines” [2].

In the same way, when symbolizing diseases, drugs are seen as markers, indications that something is not right [10,17,19,22,24,25,39], causing the experience of *adversity*. This experience is fueled by the pharmacological class and the number of drugs used concomitantly, which is expressed by both the number of pharmaceutical specialties and the dose of the drugs [10].

For many people, medications are the most visible part of health care, and the perception of their *resolution* occurs in a specific time and space, namely, Western culture of the 21st century in which medications are one of the most used technologies and a symbol of science, contributing to the unveiling of this experience [10]. It should be noted that, specifically in 2020, as a result of the coronavirus disease 2019 (COVID-19) pandemic, we have witnessed daily and fierce attempts to question the power of science, where medications have constantly been in the limelight of this discussion. It raises a question: What will be the impact of this historical process on the views of individuals about the role of medications in society?

The terms *lay pharmacology* [24] and *lay expertise* [16] are used to describe how patients construct meaning for medications and the role they play in their lives. For Cohen et al. [45], this construction occurs not only at the individual level but also at the collective level, influencing the perceptions of individuals about the daily use of medications. In the words of Merleau-Ponty [4] (p. 603): “We recognize, around our initiative and around that strictly individual project, which is oneself, a zone of generalized existence and of projects already formed, significances which trail between ourselves and things and which confer upon us the quality of man, bourgeois or worker.”

Cohen et al. [45] affirmed that medications carry meanings and are much more than just technological products or substances to be used in health care to alleviate diseases because the biological, psychological, social, economic, and cultural aspects, which are in constant interaction and evolution, affect the use of these products. According to these authors, medications must be studied as a social and cultural phenomenon.

### 2.2. Experience Related to the Use of Medications over Time

Time is one of the main structures of human existence [4]. However, Merleau-Ponty was not referring to chronological, measurable, and shared time, consisting of successive moments that are used to structure and guide daily life, but to phenomenological time [4,46,47]. Moreover, for individuals who experience a break in health, phenomenological time can be experienced as a lack of confidence about health in the future, a sense of discontinuity related to the memory of past health, and reflections on the optimal way to use time [3,6,38].

Regarding the daily use of medications, waiting time was experienced with *ambiguity* whenever it meant that time was frozen in the present [6] and patients had to wait for the proper moment to obtain an optimal response to treatment. It could also be experienced as *resolution* when it meant the possibility of keeping a routine, even if it was centered around the drug treatment [10].

The experience of illness can vary over time for the same individual. This perception can be completely different at different times, i.e., when symptoms appear, when a diagnosis is given, and several years after coexisting with a chronic illness. Furthermore, it is possible to experience periods of stability and well-being at times when illness is in the background [7].

Similarly, experience with the use of medications may also have a temporal aspect [8]. Nascimento et al. [10] described the use of antidepressants and antidiabetics, which were initially experienced as *adversity* because of the patients’ denial of the need to use the drugs. Then, after recognizing the need for the medications, and together with the fear of dependence, they experienced *ambiguity*. Finally, when medications acquired a new meaning, expressed through confidence in the treatment and certainty about the need for it, medications led to the experience of *resolution.*

One of the main contributions of these authors to the understanding of experience with the daily use of medications is the suggestion that this experience should not be understood as a constantly increasing learning curve that leads to improvement in how they are used, but as a cyclical process in which the introduction of a new drug can modify previous experiences. To explain this process, the authors used the concept of *body memory*, which Hentz [48] presented in the context of the experience of grief. This author stated that the body perceives loss, even when trying to silence it, and that the experience of grief is not a straight learning curve of overcoming and learning.

This discussion raises an important question: is cyclical temporal variation in experiences with the daily use of medications a one-off finding, or is it part of the essence of this experience? If it is an essential component, we believe it is of great importance to develop ways for health professionals to recognize how the daily use of medication is experienced. As stated by Hentz [48], recognizing *body memory* forces us to develop counseling approaches that give voice to these experiences, contributing to the implementation of truly patient-centered practice.

### 2.3. The Experience of the Other

Medications, understood as a symbol that is based on our relationship with others, reveal the disease or signal the inability of an individual to cope with life’s problems without the aid of this resource in a manner expected by society. These situations mark individuals as “different” and can cause stigma [19,20], deeply impacting social, work, and even family relationships [19].

Relationships with others that are mediated by medications can also be perceived as coercive when their use is imposed in exchange for social acceptance [19]. For some patients, taking medications is seen as a moral duty, influencing the construction of their identities and social relations [31]. For example, patients on neuroleptics see themselves as forced to use these medications by family and friends as an informal social contract such that they can be accepted by society, but they lose control of their lives and their own identity [17].

Others can also represent sources of information [18,19,20] or even support [21], influencing behaviors in relation to the use of medications. Moreover, through the establishment of therapeutic relationships, the health care professionals are an important aspect of experiences with the daily use of medications, contributing to the experience of *resolution* [10].

However, meeting with health professionals also contributed to experiences of *ambiguity* and *adversity* when individuals experienced objectification [1] or when the focus of the meetings was dysfunction, changes in laboratory tests, or changes in clinical conditions [7]. It also contributed to the perception of the absence of freedom to act because of the lack of the technical knowledge that is needed to freely choose between available treatments [6].

### 2.4. Patient Rationality in Decision-Making about Pharmacotherapy: Discussions about Sexuality and Freedom

To increase understanding of the experience of the daily use of medications, it is important to consider Merleau-Ponty’s discussion of sexuality. In the words of the philosopher, sexuality “is the general power, which the psychosomatic subject enjoys, of taking root in different settings, of establishing himself through different experiences, of gaining structures of conduct. It is what causes a man to have a history” [4] (p. 219). It can be understood as an energy that pulses and leads us to launch ourselves into life situations, to build our history in the world [4].

Coupled with the discussion of sexuality, Merleau-Ponty also discussed the issue of freedom, which is not absolute because we are situated, we are born *of* the world and *in* the world, a world that is already constituted, although not completely [4,49]. We are always in concrete situations that impose limits on our freedom, which do not determine our acts, but form the context in which we are inserted. Human freedom is “an ordinary making of choices in a world we did not choose” [49] (p. 122).

Merleau-Ponty [4] also emphasized that we are centered in the present, where our decisions come from, which are never without reason and can always be put in relation to our past. We are the ones who find a reason to act one way or the other in our conception of the past and the present, and we must refer to all of our experiences for understanding and decision-making.

Furthermore, for Merleau-Ponty [4], all the knowledge present in our consciousness has previously passed through perceptual experience, which is a bodily experience [7]. Matthews [49] (p. 33) stated that “this direct, pre-reflective, involvement is perception […], which is fundamentally a practical involvement with things. To perceive something is not just to have an idea of it, but to deal with it in some way.” Additionally, Ribeiro [50] stated that what differentiates one individual’s perception from that of another is their experience. Moreover, based on this experience, individuals make choices.

Nascimento et al. [10] reported several behaviors in the context of each of the four ways of experiencing daily medication use, namely, *resolution*, *adversity*, *ambiguity*, and *irrelevance*. These behaviors include adhering to treatment as recommended, adjusting it according to personal needs, or even discontinuing it completely. Experiencing *resolution* with the daily use of medications meant launching into life situations and acquiring structures of conduct [11], and adopting a certain position in life, which some patients called gratitude. As a result of the experience of *adversity*, participants expressed their desire not to use medications or to use fewer of them.

Ingadottir and Halldorsdottir [20] (p. 612) stated that “to do right is a strong element in human beings, but what counts as right isn’t always clear,” with conflicting desires occurring all the time. People are constantly negotiating with themselves in an attempt to lead a normal life.

Faced with the need to use medications persistently as a strategy for coping with chronic disease, they develop several behaviors in response to this demand. Furthermore, whether consciously or not, they formulate hypotheses about the effects of the medications, testing them by interrupting them, by reducing doses, and even by giving themselves “vacations” to evaluate the effectiveness and/or reduce the toxicity [19,33,34]. Some patients argue that complete adherence is not necessary to obtain the maximum effect and that flexibility allows for control over their lives. Others assert that total adherence is not possible [18]. Blaxter and Britten [51] conducted a review of lay beliefs about drug use that came to a perplexing conclusion: individuals do not see drugs as something to be taken as prescribed, but rather as something to be used when they deem appropriate.

For diabetic patients, the *right thing to do* is related to that which causes physical and mental well-being and balance, which often means partially or completely changing medication regimens, to the detriment of the risk of complications [21,22,32]. About 80% of psychiatric patients change their treatment at some point, resulting in uncontrolled illness [17]. Pound et al. [19] also reported that patients with hypertension or those who use medications such as neuroleptics change the dose or stop taking the drug to ingest alcoholic beverages. A significant proportion of hypertensive older adults reported that they adhered to treatment but they made it clear that they did not follow the medical prescription correctly, making changes to the dosage, for example, [52], which leads us to the following question: what concept do patients have about what it means to follow a treatment correctly?

For patients with thalassemia, the impact of weekly absences from work as a result of treatment compromise patients’ employment and source of income. Irregularities in treatment are not attitudes of rebellion or ignorance, but an expression of the need to normalize the body and life. However, the biggest issue for health professionals is keeping patients alive. In the words of the author, “While everyone focuses on the same problem, each person has a different emphasis on its meanings. This creates oppositions and generates different priorities” [27] (p. 7).

When coping with stigma, patients with HIV/AIDS [25] or hepatitis C [53,54] seek to keep the disease secret and separate from other parts of their lives, which can compromise the use of medications and the effectiveness of treatment. Moreover, patients with asthma reject the use of inhaled corticosteroid drugs, which are necessary to control the disease, because this means admitting its presence [19].

Ingadottir and Halldorsdottir [21] found that knowledge, understanding, and experience created the basis for adherence to treatment, but they may not be sufficient because even conscientious patients chose not to adhere entirely to treatment, viewing it as a nuisance. Nonadherence was considered a very complex phenomenon that is also associated with strategies regarding self-protection and maintaining quality of life in light of what the participants considered inadequate clinical decisions.

All these adjustments made by individuals to their drug therapies are essential for them to truly understand the role of medications in their new life context, i.e., in the presence of chronic diseases. A study by Pound et al. [19] went a step further and stated that professionals should help patients take the necessary tests responsibly such that they can actually adhere to the treatments. Shoemaker and Ramalho de Oliveira [9] argued that patients are able to assess the risk–benefit ratios of treatments and to decide whether to continue using a drug, as long as they believe that the benefits outweigh the risks.

However, in the paternalistic model of the physician–patient relationship, patients are seen as people who do not contribute any expertise to the relationship, which underestimates the constant decisions and adjustments they need to make in their drug therapies [45]. The author addressed this issue in a very revolutionary way by stating that the paradigm of the rational use of medications is limiting and inadequate to understand the role they play in society. Pound et al. [19] stated that patients have logical reasons not to use the prescribed medications and that an ideology of power is associated with the concepts of compliance, agreement, and adherence.

Nonadherence can be seen as a deliberate strategy by patients to assert their autonomy, negotiate with doctors, and reject the disability attributed to the use of drugs [45]. These different ways of dealing with pharmacological treatments demonstrate that patients are active agents in their treatment [24]. Moreover, as stated by Shoemaker and Ramalho de Oliveira [9], taking control is a key theme in the subjective experience of patients with chronic drug use. Pound et al. [19] added elements to this discussion by proposing that the term *resistance* is suitable for the evaluation of how patients use drugs: they take them but try to minimize their use. These authors also stated that this term can also be applied to relationships that involve the exercise of power and coercion, pointing to relationships established within the context of health care.

Wong and Ussher [18] argued that there is an existential aspect to the issue of adherence, which refers to responsibility where individuals are responsible for creating their own lives, choices, and actions. Furthermore, for Usher [17], the place given by patients to treatment in their lives is related to adherence or the lack thereof; a preponderant factor in adherence is having a productive present and even a future.

Contributing to this discussion, Ratcliffe [55,56] defined the spaces of possibilities that underlie our sense of belonging to the world as “existential feelings.” The author emphasized that existential feelings consist not of “an abstract, static sense of the possible,” but of an anticipatory structure that permeates a person’s involvement with the world, which is capable of opening or closing possibilities of experiences. Moreover, in this regard, they can be used as a lens through which we interpret various experiences [56].

In a study conducted by Nascimento et al. [10], some patients tried to re-signify the use of medications in their lives, ceasing to see them as *enemies* and establishing a relationship of *friendship*. Others used religiosity to express the meaning of medications in their lives, something that is both divine and demonic. Furthermore, in this context, feelings, such as gratitude, faith, hope, and fear, can be understood as conditions for the possibility of experiencing the daily use of medications [10]. In corroboration, Ridgeway et al. [42] reported that developing coping strategies, including religiosity and faith, in addition to focusing on life priorities, planning for the future, and maintaining positive attitudes, are important for reducing the burden of treatment, including pharmacological burdens. Wong and Usher [18] described the narrative of three HIV/AIDS patients who experienced adverse reactions. For each patient, this experience carried a different weight, which was related to the place given to the treatment in their lives.

Once again, important questions emerge. How can we apprehend existential feelings in clinical practice? How do they manifest in the experience of medication use? Considering medications as conditions of possibility that can either open or close possibilities of experiences, is there some way they can be modified and/or leveraged in the clinical context with the aim of improving the experience of patients and health outcomes?

This manuscript, throughout the use of theory, presented the medication experience as a rich concept that can and should be operationalized in the practice of the pharmacist. The medication experience is a real, powerful, and embodied experience that is lived by individuals during the use of medications. Patients experience medications in different ways, which affect their decision-making processes, their behaviors, and consequently, their health outcomes.

Thus, it is our understanding that in order to practice in a patient-centered manner, pharmacists should take these experiences into consideration in their interaction with every patient when using any type of medication and with any health condition. All things considered, the questions summarized in Table 2 can help clinical pharmacists in this task.

## 3. Final Considerations

We believe that because it is anchored in the phenomenal body, the phenomenology of Merleau-Ponty is an important theoretical framework for the study of experience related to the daily use of medications. Moreover, the unveiling of the four ways of experiencing this use—*resolution*, *adversity*, *ambiguity*, and *irrelevance*—is a product of the application of this theoretical framework. This is especially true when explaining the forms *ambiguity* and *irrelevance*, which are often neglected in studies that evaluate only the positive and negative aspects of medication use.

However, some issues raised throughout this article remain open and invite us to explore them, such as (1) the coexistence of multiple ways of experiencing the use of medications, by the same individual, in a given historical time; (2) the cyclical structure of this experience; (3) the impact of habit and routine on the ways of experiencing the daily use of medications; and (4) the contribution of the concept of existential feelings to this experience and its impact on decision-making by patients.

Throughout this discussion, we realized that the experience with the daily use of medications is a complex and multifaceted phenomenon that directs the decision-making process of patients, impacting health outcomes. Furthermore, because it has profound implications for clinical practice, we need to broaden the understanding of this phenomenon and its dissemination through teaching future healthcare professionals and facilitating its application in healthcare environments.

## Figures and Tables

**Table 1:** Ways of experiencing the use of medicines.

Ways of Experiencing the Use of Medicines	Elements Linked to These Experiences
*Resolution*	Interruption of the evolution of the disease
	Prevention of harm
	Reduction of symptoms
	Improvement of clinical parameters (predictability)
	Normalization of life
*Adversity*	Occurrence of adverse drug reactions and the fear of adverse reactions
	Difficulties inherent to the use of the drug
	Difficulty in achieving the goal of therapy
	Fear of stigma and discrimination
	Fear of dependence
	Fear of a lack of effectiveness and waiting time for effectiveness
	Fear of changes in life projects
	Symbol of trouble, that something is not right
	Feeling of incoherence between the markers of the disease and the perception of their health and well-being
*Ambiguity*	Finding a balance between acknowledging there is a need for the drug and the fear of taking it
	Requirement of the acquisition of new habits
*Irrelevance*	There is no change in the phenomenal/lived body caused by the disease or the medications

**Table 2:** Summary of aspects about the patient’s medication experience that health practitioners should pay attention to in clinical practice.

Aspects of the Medication Experience	Related Questions
Ways of experiencing	How have your patients been experiencing the daily use of medications?
	Are there different ways to experience this at the same time in life, depending on the disease and the drug in question?
	How can you use this knowledge to implement/provide a more patient-centered service?
Habits and routines	What is the impact of habits and routines on how your patients experience the daily use of medications?
	How can you identify individuals who are overwhelmed by the burden of adhering to complicated treatment routines?
	How can you provide the conditions to integrate drugs into your patient’s daily life and help to harmonize individuals and their treatment?
Temporal aspects	Do you recognize that your patient’s experience can vary with time?
	How can you use the knowledge about *body memory* and cyclical temporal variation to build a more patient-centered practice?
The role of the healthcare professional	How can health professionals minimize the experiences of *ambiguity* and *adversity* in clinical practice?
	How can health professionals foster positive medication experiences, such as *resolution*?
Existential feelings	How do existential feelings impact the experience of the daily use of medication?
	Is there some way that existential feelings can be modified in the clinical context with the aim of improving the experience of patients and their health outcomes?

## Data Availability

Not applicable.
